# *In silico* analysis of protein Lys-N^𝜀^-acetylation in plants

**DOI:** 10.3389/fpls.2014.00381

**Published:** 2014-08-04

**Authors:** R. Shyama Prasad Rao, Jay J. Thelen, Ján A. Miernyk

**Affiliations:** ^1^Division of Biochemistry, University of MissouriColumbia, MO, USA; ^2^Interdisciplinary Plant Group, University of MissouriColumbia, MO, USA; ^3^Plant Genetics Research Unit, United States Department of Agriculture – Agricultural Research ServiceColumbia, MO, USA

**Keywords:** acetyltransferase, bromodomain, deacetylase, interactome, post-translational modification, regulation, signaling

## Abstract

Among post-translational modifications, there are some conceptual similarities between Lys-N^𝜀^-acetylation and Ser/Thr/Tyr O-phosphorylation. Herein we present a bioinformatics-based overview of reversible protein Lys-acetylation, including some comparisons with reversible protein phosphorylation. The study of Lys-acetylation of plant proteins has lagged behind studies of mammalian and microbial cells; 1000s of acetylation sites have been identified in mammalian proteins compared with only hundreds of sites in plant proteins. While most previous emphasis was focused on post-translational modifications of histones, more recent studies have addressed metabolic regulation. Being directly coupled with cellular CoA/acetyl-CoA and NAD/NADH, reversible Lys-N^𝜀^-acetylation has the potential to control, or contribute to control, of primary metabolism, signaling, and growth and development.

## INTRODUCTION

Protein regulation is highly choreographed, and encompasses multilayered and interconnected transcriptional and translational processes. Subsequent PTM allow the fine-tuning of function. Many PTM are reversible, and modulate the activity, localization, turnover, and interactions of proteins (Mann and Jensen, 2003). The dynamics of PTM allow a much more rapid response to cellular signals than transcriptional or translational regulation.

More than 461 distinct PTM have been described^1^ (Khoury et al., 2011), and it is increasingly clear that many, perhaps most, proteins are decorated with multiple PTM (Hunter, 2007). This yields a proteome vastly larger and more diverse than the translated genome (Khoury et al., 2011; Minguez et al., 2012). The combinatorial diversity of PTM results in enormous flexibility for control of structure, function, and interaction. For example, Ser/Thr/Tyr-phosphorylation introduces a negative charge while Lys-N^𝜀^-acetylation neutralizes a positive charge.

## REVERSIBLE O-PHOSPHORYLATION

Reversible protein phosphorylation was first reported in 1954, and has remained the hallmark of PTM-based regulation and signaling (Pawson and Scott, 2005). Approximately 130 protein kinases are encoded by the *Saccharomyces cerevisiae* genome, 518 in *Homo sapiens*, 1085 in *Arabidopsis thaliana*, and as many as 2500 in some land plants (Lehti-Shiu and Shiu, 2012). In yeast there are >2500 known phosphoproteins (P-proteins; Rao and Møller, 2012), more than 31,480 phosphosites (P-sites) have been described in 7280 *Rattus norvegicus* proteins (Lundby et al., 2012b), and ∼14000 P-sites in >4000 proteins have been described from the reference eudicot plant *A. thaliana*^2^. Considering all plants, these numbers swell to ∼33,300 sites on ∼11,700 P-proteins^2^, and more are discovered virtually daily. The recent reports of thousands of Lys-N^𝜀^-acetylation sites in bacterial, yeast, and animal proteins have led to the suggestion that this PTM could be as common as phosphorylation (Kouzarides, 2000; Choudhary et al., 2009; Hu et al., 2010; Filippakopoulos and Knapp, 2012; Lundby et al., 2012a,b; Xiong and Guan, 2012). An overview comparison between these two major systems for PTM is presented in **Table 1**.

**Table 1:** The components of the Ser/Thr/Tyr O-phosphorylation and Lys-N^𝜀^-acetylation systems from *Homo sapiens*, *Saccharomyces cerevisiae*, and *Arabidopsis thaliana*.

	Phosphorylation		Acetylation	
	Number	Reference	Number	Reference

	Phospho-proteins (sites)			Acetylated-proteins (sites)
*H. sapiens*	19800 (208500)	www.phosphosite.org	2859 (5011)	Choudhary et al. (2009), Kim et al. (2006), Zhao et al. (2010)
*S. cerevisiae*	2552 (11481)	Rao and Møller (2012)	1059 (4000)	Henriksen et al. (2012)
*A. thaliana*	4081 (13933)	http://p3db.org	221 (329)	Finkemeier et al. (2011), König et al. (2014a) Wu et al. (2011)
				
	Kinases		Acetyltransferases	
*H. sapiens*	518	Manning et al. (2002)	22	Xiong and Guan (2012)
*S. cerevisiae*	130	Breitkreutz et al. (2010)	11	Yang (2004b)
*A. thaliana*	1085	Hrabak et al. (2003)	16	Earley et al. (2007), Tyler et al. (2006), http://uniprot.org/
				
	Phosphatases		Deacetylases	
*H. sapiens*	147	Moorhead et al. (2007)	18	Xiong and Guan (2012)
*S. cerevisiae*	38	Breitkreutz et al. (2010)	4	Bernstein et al. (2000)
*A. thaliana*	150	Kerk et al. (2008)	18	Hollender and Liu (2008)
				
	SH2-/14-3-3-proteins		YEATS-/BRD-proteins	
*H. sapiens*	111/207	Liu et al. (2011) Tinti et al. (2012)	4/46	Schulze et al. (2009) Filippakopoulos et al. (2012)
*S. cerevisiae*	1/2	Liu et al. (2011) van Heusden and Steensma (2006)	3/9	Schulze et al. (2009) Zhang et al. (2010)
*A. thaliana*	2/13	Williams and Zvelebil (2004) DeLille et al. (2001)	2/29	Zacharaki et al. (2012) http://supfam.cs.bris.ac.uk

## REVERSIBLE Lys-N^𝜀^-ACETYLATION

There are multiple forms of protein acetylation; O-acetylation of Ser/Thr residues, non-reversible N^α^-acetylation of protein N-termini (Martinez et al., 2008), and reversible protein N^𝜀^-acetylation of internal Lys residues (Soppa, 2010; Xing and Poirier, 2012). While important in their own right, the first two types of Lys-acetylation will not be further addressed herein. Instead, “acetylation” should be understood to mean protein Lys-N^𝜀^-acetylation (PKA). Histones were first reported to be acetylated nearly 50 years ago (Phillips, 1963). It was more than two decades later that the first cytoplasmic protein, α-tubulin, was identified as being Lys-acetylated (Piperno and Fuller, 1985). Recently, a plethora of acetylated proteins have been described encompassing all subcellular compartments (Choudhary et al., 2009; **Table 2**). During these few years, PKA has changed from an obscure histone PTM to a mechanism for controlling (or contributing to control of) many aspects of primary metabolism, gene expression, signaling, and development (Zhao et al., 2010; Rao et al., 2014).

**Table 2:** Identification of Lys-N^𝜀^-acetylated proteins in various taxa.

Taxa	Proteins	Sites	Reference
**Plants**			
*A. thaliana*	74	91	Finkemeier et al. (2011)
	90	174	König et al. (2014a)
	57	64	Wu et al. (2011)
*G. max*	121	190	Smith-Hammond et al. (2014b)
*P. sativum*	358	664	Smith-Hammond et al. (2014a)
*Vitis vinifera*	97	138	Melo-Braga et al. (2012)
*S. tuberosum*	31	35	Salvato et al. (2014)
			
**Animals**			
* H. sapiens*	1750	3600	Choudhary et al. (2009)
	>62	>111	Kim et al. (2006)
	1047	>1300	Zhao et al. (2010)
*Mus musculus*	133	277	Kim et al. (2006)
*R. norvegicus*	4541	15474	Lundby et al. (2012a)
			
**Yeast**			
* S. cerevisiae*	>1059	>4000	Henriksen et al. (2012)
			
**Protists**			
* P. falciparum*	230	421	Miao et al. (2013)
*T. gondii*	274	411	Jeffers and Sullivan (2012)
			
**Archaea**			
* Halobacterium* sp.	2	2	Soppa (2010)
			
**Bacteria**			
* E. coli*	85	125	Yu et al. (2008)
	91	138	Zhang et al. (2009)
	349	1070	Zhang et al. (2013)
*S. enterica*	191	>191	Wang et al. (2010)
*B. subtilis*	185	332	Kim et al. (2013)

Use of high-throughput, high-resolution tandem MS has led to detection of PKA in all three domains of life; archaea, bacteria, and eukaryotes (Yang, 2004a; Hu et al., 2010; Soppa, 2010; Jones and O’Connor, 2011). Recent MS-based studies revealed 1070 acetylation sites in 349 proteins in *Escherichia coli* (Zhang et al., 2013), and 332 acetylation sites on 185 proteins in *Bacillus subtilis* (Kim et al., 2013). In *S. cerevisiae*, more than 4000 sites of Lys-N^𝜀^-acetylation of 1059 proteins have been described (Henriksen et al., 2012), and in mammalian systems >21,000 sites on >7000 proteins have been described (Choudhary et al., 2009; Zhao et al., 2010; Lundby et al., 2012a). A large majority of the yeast and mammalian Lys-acetylation sites have been described since 2009 (Choudhary et al., 2009; Smith and Workman, 2009), suggesting that our understanding and appreciation for this PTM is in a relatively early stage especially in comparison with phosphorylation ().

The results from large-scale secondary structure analyses have led to the conclusion that O-phosphorylation is substantially enriched in regions of ISD (Iakoucheva et al., 2004). It has been reported that PKA sites are significantly enriched in ordered regions of mammalian proteins and depleted in regions of ISD (Choudhary et al., 2009), that PKA sites are equally distributed in ordered and disordered regions (Gao and Xu, 2011), and that PKA sites preferentially occur in regions of ISD in *Toxoplasma gondii* tachyzoite proteins (Xue et al., 2013). We observed that versus all Lys residues in our soybean database, PKA was approximately twice as likely to occur in long ISD-regions (Smith-Hammond et al., 2014b). If, as has recently been proposed (Cumberworth et al., 2013), regions of ISD are important in mediating protein interactions, then PTM of residues within regions of ISD might explain the basis for multiple layers of regulation (Nishi et al., 2013). Clearly this aspect of PKA deserves a greater focus.

## Lys-N^𝜀^-ACETYLATION IN PLANTS

While thousands of acetylated proteins are known in animals and yeast, when preparing this manuscript fewer than 400 plant proteins (∼500 sites) have been reported. These include 131 *A. thaliana* proteins (155 sites; Finkemeier et al., 2011; Wu et al., 2011), 97 *Vitis vinifera* berry proteins (138 sites; Melo-Braga et al., 2012), 121 proteins (190 sites) from developing *Glycine max* cotyledons (Smith-Hammond et al., 2014b), and 31 proteins (35 sites) from *Solanum tuberosum* mitochondrial proteins (Salvato et al., 2014). Even after results from work in progress are added; 90 *A. thaliana* mitochondrial proteins (174 sites; König et al., 2014a), and 358 *Pisum sativum* proteins (664 sites; Smith-Hammond et al., 2014a), the extant number of PKA proteins is small in comparison to P-proteins and only five species have been examined.

## “WRITERS, ERASERS, AND READERS” OF Lys-N^𝜀^-ACETYLATION

The occurrence of a mammalian enzyme specific for histone acetylation was first reported by Racey and Byvoet (1971), and the first histone acetyltransferase (HAT1) gene was cloned from yeast (Kleff et al., 1995). For over 40 years, protein Lys-acetylation has meant “histone Lys-acetylation.” The relatively recent discovery that transcription factors, structural proteins, metabolic enzymes, and a host of other non-histone proteins are Lys-N^𝜀^-acetylated has led to some confusion about specificity and terminology (Josling et al., 2012; Xing and Poirier, 2012). There are even instances where a “HAT” has been reported to acetylate histones, non-histone proteins, and even small molecules (Gu and Roeder, 1997). At this point, we favor erring on the side of generalization rather than claiming unsupported specificity. With this caveat, herein we refer to the “writers” of the PTM code (Muntean and Hess, 2009), protein Lys-N^𝜀^-acetyltransferases, as KATs, and the “erasers,” protein Lys-N^𝜀^-deacetylases as KDACs.

**FIGURE 1:**
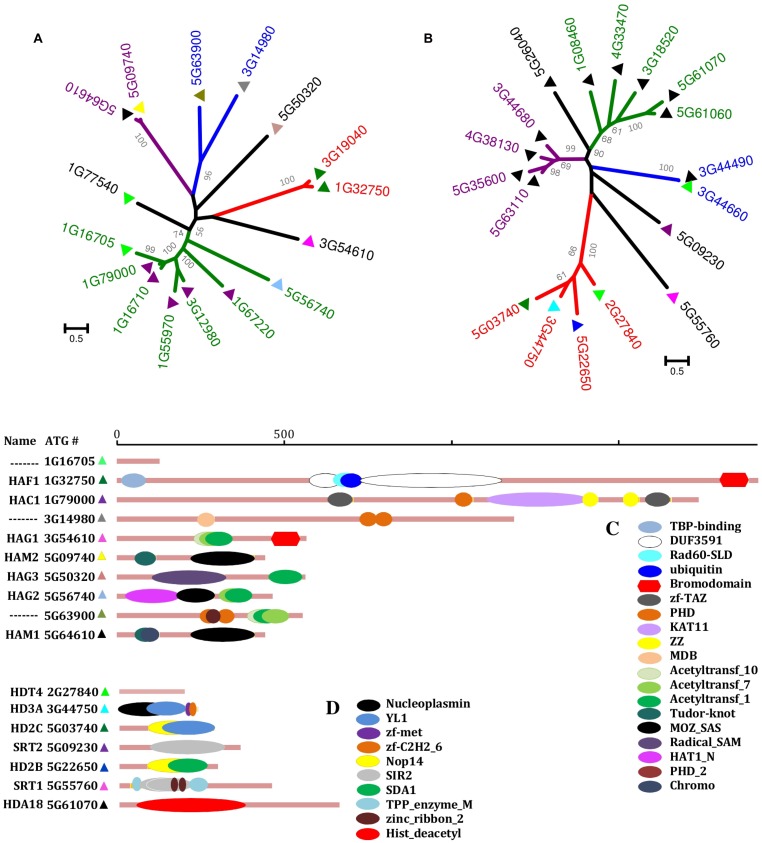
**Phylogeny and domain architecture of the *Arabidopsis thaliana* KATs and KDACs.** The phylogenetic trees of KATs **(A)** and KDACs **(B)** are shown with distinct groups colored differently. The KAT and KDAC sequences were aligned using MUSCLE, and trees were constructed in MEGA5 (http://www.megasoftware.net/) using maximum-likelihood method with 1000 bootstrap replicates (Values of more than 50% are shown). Scale bars indicate the number of substitutions per site. For the domain architecture of selected KATs **(C)** and KDACs **(D)**, information was collected from PFAM (http://pfam.sanger.ac.uk/). Horizontal bars are proportional to sequence length (129–1919 residues for KATs, 203–682 residues for KDACs).

Based upon comparative sequence analyses, it has been concluded that there are four distinct KAT families: GNAT (GCN5-related N-terminal acetyltransferases); MYST [**M**OZ, **Y**bf2 (Sas3), **S**as2, and **T**ip60; Sapountzi and Côté, 2011]; p300/CREB (CBP); and the nuclear receptor coactivator family (Roth et al., 2001). The first three families are widespread in eukaryotes [including *A. thaliana* (Pandey et al., 2002)], eubacteria, and archaea. The nuclear receptor coactivator family is thought to be absent from plants, fungi, and lower animals (Pandey et al., 2002). The different KAT families have distinct kinetic and catalytic mechanisms (Yan et al., 2002; Röttig and Steinbüchel, 2013), but are characteristically large, complex, multi-domain proteins (Roth et al., 2001; **Figures 1A,C**).

Perhaps not all protein acetylation is KAT-mediated? Similar to non-kinase based protein phosphorylation (autophosphorylation; Miernyk et al., 1992; Bae and Schlessinger, 2010), there have also been reported examples of autoacetylation (e.g., Yang et al., 2012). An intriguing recent publication described “widespread and enzyme-independent N^𝜀^-acetylation” of a number of proteins in the mitochondrial matrix (Wagner and Payne, 2013). This mechanism might also be extended to include peroxisomal and plastidial proteins? It is noteworthy that König et al. (2014b) found N^𝜀^-acetylated proteins, and a sirtuin, within *A. thaliana* mitochondria, but found no evidence of a KAT. Nor was there any evidence for a KAT within highly purified potato tuber mitochondria (Salvato et al., 2014).

If the KATs write the PTM code, then it is the deacetylases that are responsible for “erasing” it. The genome of the reference eudicot plant *A. thaliana* includes 18 genes encoding KDAC proteins; at least two members each of the RPD3-like (**r**educed **p**otassium **d**eficiency 3), HD-tuin, and sirtuin families (Hollender and Liu, 2008). Much like the KATs, the KDAC proteins display complex domain organization (**Figures 1B,D**), tissue-specific expression, and physiological functions. Members of the RPD3-like family are apparently present in all eukaryotes and have been the most widely studied KDACs (Murfett et al., 2001; Rossi et al., 2003). The HD-tuins appear to be present only in plants (Dangl et al., 2001; Luo et al., 2012) and have been the least studied.

The sirtuins (Silent Information Regulator 2 proteins) are a ubiquitous family of NAD^++^-dependent KDACs. It has been reported that mammalian cells contain seven sirtuin homologs (SIRT1–7) with diverse cellular localization [for example, some proteins of the Srt3, 4, and 5 families are mitochondrial (Huang et al., 2010; Sol et al., 2012)] and physiological functions. The sirtuins have been demonstrated to deacetylate a wide spectrum of clients (Finkemeier et al., 2011; Duan, 2013). König et al. (2014b) recently described a Srt2-orthologous protein localized within the matrix of *A. thaliana* mitochondria that deacetylates a specific cohort of mitochondrial client proteins. In contrast to the seven sirtuin genes present in mammalian genomes, *A. thaliana* and *G. max* (Glyma04g38730.1 and Glyma06g16260.1) have only two sirtuin-encoding genes. König et al. (2014b), however, detected more than seven alternative splicing variants of Atsrt2.

**FIGURE 2:**
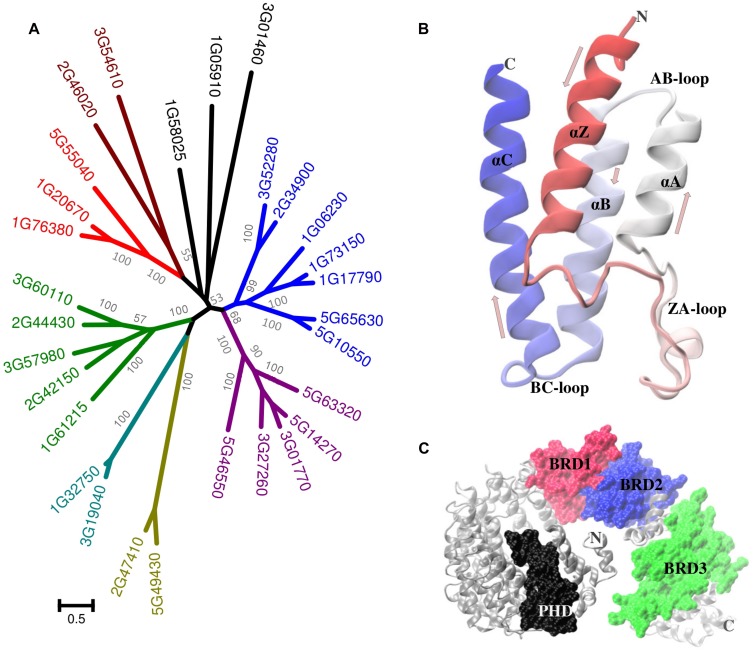
**Plant bromodomain proteins. (A)** Phylogenetic analysis of the *A. thaliana* BRD-proteins. Distinct clusters (more than 50% bootstrap value) are colored differently in the tree. Sequences were aligned with MUSCLE and the tree was constructed using maximum likelihood method with 1000 bootstraps in MEGA5.The scale bar indicates the number of substitutions per site. **(B)** A representative BRD structure (AT3G54610.1) has four helices (Z, A, B, and C, each ∼110 residues in length). The ZA plus BC loops form the acetyl-Lys binding pocket. Unlike many mammalian and yeast proteins which include two or more BRD, most plant proteins have a single BRD. **(C)** An exception is the product of the *Chlamydomonas reinhardtii* (gi| 159485810) locus which includes three BRD (plus a PHD domain). The *C. reinhardtii* protein structure was predicted using I-TASSER ( ).

Like a molecular barcode, the information present in acetylated-Lys must be recognized and decoded by some sort of “reader.” Originally discovered as a component of histone-binding proteins, bromodomains (BRD) are conserved structural motifs (**Figure 2B**) that recognize and bind PKA (Dhalluin et al., 1999; Zeng and Zhou, 2002; Sanchez and Zhou, 2009). The term “BRD” comes from brahma, a regulatory protein in *Drosophila melanogaster*. The human genome encodes at least 46 BRD-proteins (each of which has between one and six BRDs) which have been sorted into eight classes (Filippakopoulos et al., 2012). The yeast genome encodes at least nine BRD-proteins (Sanchez and Zhou, 2009; **Table 1**).

**FIGURE 3:**
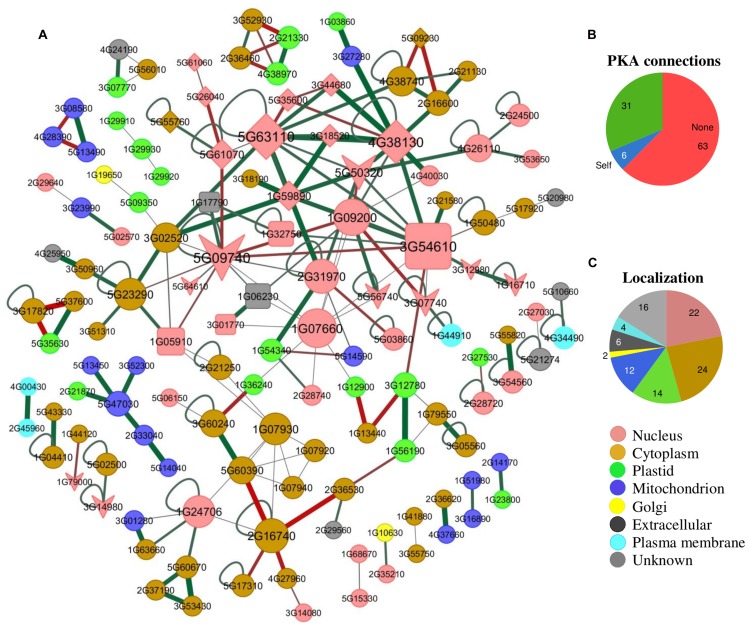
**A plant Lys-N^𝜀^-acetyl-protein interactome. (A)** The BRD-proteins are presented as squares, KATs as arrow-heads, KDACs as diamonds, and PKA proteins as circles. Some of the PKA proteins [histone H4 (AT1G07660) and H3.2 (AT1G09200), GTP-binding EF-Tu (AT1G07930), and nuclear PGK1 (AT3G12780)] interact with BRD-proteins. However, either no interactions or only self-interactions are known for the majority of plant PKA-proteins (∼69%; **B)**. The interactome information was collected from TAIR (), AtPID (), AtPIN (), CCSB ( ), and PAIR (), and visualized using Cytoscape. Edge color (red is negative and green is positive) and width are proportional to gene co-expression (correlation coefficient) of genes based on GSE3011 from NCBI-GEO (). Nodes with no known interactions have not been included. Node size is proportional to the number of interactions. Node colors indicate subcellular protein localization, based on information from the TAIR database. **(C)** The percentage of currently known plant PKA-proteins in different subcellular localization categories is shown. The *A. thaliana* homologs of known PKA-proteins from other plants species were obtained using BLAST.

In *A. thaliana* there are 29 BRD-proteins^3^, which can be separated into multiple groups (**Figure 2A**). The number of BRD-proteins varies considerably among plants, from as many as 57 in *G. max* to as few as nine in the red nanoalga *Cyanidioschyzon merolae.* There are only a few instances of plant proteins that include more than a single BRD (**Figure 2C**). The relationship between BRDs and Lys-acetylated client proteins (**Figure 3**) has been compared with the recognition and binding of O-phosphorylated client proteins with the SH2 domain or with 14-3-3 proteins (Yang, 2004a; de Boer et al., 2013). It is not yet clear if recognition and binding involve only acetylated-Lys residues or if these residues must be in a particular context/domain/environment.

An additional possible PKA-reader is the YEATS domain, which is an evolutionarily conserved structural feature found in a variety of proteins in chromatin-modifying and transcription complexes ranging from yeast to humans (Schulze et al., 2010). Recently, the 3D structure of the YEATS domain from Yaf9 protein has been resolved, which is shown to have a region containing a shallow groove that might constitute aPKA-binding pocket (Zhang et al., 2011). The YEATS-domain containing protein family comprises more than 100 members in over 70 eukaryotic species (Schulze et al., 2009), including *A. thaliana* (Zacharaki et al., 2012), *Oryza sativa*, and *Medicago truncatula*.

It is worthwhile to note that not all effects of acetylation related downstream functions require BRD proteins, or any kind of mediating protein binding. Just as with O-phosphorylation, acetylation can exert direct effects on protein function or enzyme activity. For example, in *Escherichia coli* 85 PKA proteins (125 sites) have been identified (Yu et al., 2008), which seemingly function in the absence of any known BRD-proteins^3^.

Although the total number of P-sites and PKA sites are converging (**Table 2**), there remain large differences in the numbers of components of each system. While there are hundreds of Ser/Thr/Tyr-kinases (518/130/1085 in *H. sapiens*/*S. cerevisiae*/*A. thaliana*) and phosphatases (147/38/150), there are relatively few KATs (22/11/16) and KDACs (18/4/18). How can so few enzymes specifically regulate thousands of acetylation events in a cell (Kouzarides, 2000; Xing and Poirier, 2012; Xiong and Guan, 2012)? How does PKA accomplish specific and dynamic control with a relatively small complement of writers, erasers, and readers? Choi and Bakkenist (2013) recently suggested an intriguing possibility, that a PTM-binding protein might function in part by blocking access of a kinase and/or acetyltransferase to a different, potentially regulatory-site. Additionally, there are several reports that both KAT and KDAC are present *in vivo* as components of multi-protein complexes. Associated proteins can both stimulate (Berndsen et al., 2008) and inhibit (Kim et al., 2014) acetyl-transferase activity, and might also specify or modify client protein selectivity.

The known writers, erasers, and readers are all relatively large, multi-domain proteins (**Figure 1**). Multiple domains often function as protein interaction modules or scaffolds. This implies a profusion of protein interactions, multi-protein complexes, and networks (**Figure 3**). With the exception of BRD-proteins binding to PKA-histones (Dhalluin et al., 1999), most protein interactions have been addressed from a computational perspective (Lu et al., 2011). Protein interactions, signaling networks, etc. have now been well-studied as related to reversible O-phosphorylation (Trost et al., 2010; Newman et al., 2013; Uhrig et al., 2013), and it will be important to extend our understanding of PKA in this direction.

## Lys-N^𝜀^-ACETYLATION AND METABOLIC CONTROL

Lys-acetylation has rapidly become established as an important PTM involved with metabolic regulation in mammalian and microbial systems. In yeast and mammalian systems, virtually every enzyme of glycolysis, gluconeogenesis, the Krebs cycle, and urea, fatty acid, and glycogen metabolism has been reported to be acetylated (Wang et al., 2010; Zhao et al., 2010; Oliveira and Sauer, 2012). However, distinguishing *bona fide* regulatory sites among the thousands of PKA sites detected by contemporary high-resolution MS, and elucidating the mechanisms by which the modifications alter protein function remain a primary challenge.

The multiple mechanisms by which reversible-O-phosphorylation can directly affect protein activity includes effects on catalysis, the binding or release of substrates/products/regulators, protein complex formation, localization, turnover, etc. (Zhang et al., 2007; Trost et al., 2010; Oliveira et al., 2012; Fischer, 2013). As a rule of thumb, a PTM directly involved with control of protein function will be dynamic (e.g., have a shorter lifetime than the protein itself). A successful experimental strategy has been to use phosphatase or deacetylase inhibitors to treat tissues, cells, or organelles. Alternatively, it is also useful to employ knockout or knock-down phosphatase or deacetylase mutants. Finally, it is possible to use recombinant phosphatases or deacetylases to treat modified proteins coupled with direct measurements of activity.

While our current understanding of reversible PKA in plants is both preliminary and fragmentary, there is support for regulation in a few instances. Deacetylation of 3PGA-kinase by incubation with a heterologous sirtuin led to a significant increase in catalytic activity (Finkemeier et al., 2011). Likewise, RuBisCO LSU is Lys-acetylated *in vivo*, which reduces activity (Finkemeier et al., 2011). There are several reports that PKA inhibits/prevents/reverses protein interactions. Plastidial glycolytic/Calvin cycle enzymes form a complex/metabolon in the stroma (Graciet et al., 2004) mediated by PKA of GAPDH (Winkel, 2004). The position and acetylation of the specific GAPDH Lys residue are conserved in animal and bacterial sequences (Zhao et al., 2010; Zhang et al., 2013), as is, presumably, the role in mediating protein interactions. Another example of PKA affecting protein interactions is LHC subunit trimer formation and association with the thylakoid membranes (Wu et al., 2011). Unfortunately, thus far there have not yet been many instances where protein interactions have been directly related to protein activity.

## Lys-N^𝜀^-ACETYLATION AND SIGNALING

Changes in the complex pattern of histone PKA as a mechanism for controlling gene expression is being studied extensively (e.g., Cigliano et al., 2013; Gu et al., 2013), and is not addressed herein. Instead, we will focus on signaling between the nucleus and the cytoplasmic organelles. Plastids and mitochondria are specialized for both production and utilization of ATP and reduced pyridine nucleotides. While both plastids and mitochondria are “semi-autonomous,” the vast majority of proteins resident in these organelles is nuclear-encoded, translated in the cytoplasm, and imported post-translationally. The protein complements of these organelles are dynamic, and must be regulated to match cellular energy demands. Mechanisms for this regulation include sensing metabolic states and signaling the nucleus of changes.

Nuclear regulation of organellar protein composition and concentration is extensive and complex, and signaling is considered anterograde or forward. However, the idea of organellar signaling leading to changes in nuclear gene expression is a newer concept and is referred to as retrograde signaling (Ng et al., 2014). While our understanding of retrograde signaling is at a very early nascent stage, it has nevertheless been surprising that the best understood signaling molecules are simple metabolic intermediates (e.g., Czarnecki et al., 2012).

In a remarkable example of both flexibility and economy, acetyl-CoA is both a central metabolite and the substrate for PKA (Hartl and Finkemeier, 2012; Wellen and Thompson, 2012). Acetyl-CoA is a key component of major metabolic pathways in the cytoplasm, peroxisomes, plastids, and mitochondria, and examples of PKA of enzymes have been identified in all of these subcellular compartments (Finkemeier et al., 2011; Wu et al., 2011). Thus the metabolic status of these organelles, and of the cell in general, might easily be signaled to the nucleus. In order to maintain compartmental specificity, it would be necessary that the signaling molecules be either an up- or downstream component of the specific pathways. It has been recently suggested that citrate and possibly malate could be the signals for mitochondrial retrograde signaling (Finkemeier et al., 2013).

Another potentially important aspect of PKA and signaling involves subcellular dynamics. Both organelles and cytoplasmic protein complexes employ molecular motors to move along the cytoskeleton. Actin filaments, intermediate filaments, and microtubules are all subject to PKA, which can affect both intracellular trafficking and protein interactions (Zencheck et al., 2012). Cytoplasmic GAPDH can function either as part of a soluble, non-associated glycolytic pathway or as a component of a glycolytic metabolon at the mitochondrial outer membrane (Graham et al., 2007). All aspects of this dynamic microcompartmentation are potentially controlled by PKA, including association of the GAPDH subunits, association with the metabolon (several if not all components of which are subject to PKA) and positioning in the cell via association with actin (Wojtera-Kwiczor et al., 2013). It seems reasonable to assume that the metabolic signals sent to the nucleus would differ under each of these conditions. Finally, it is important to consider interactive and hierarchical interactions among PKA, other PTM, and other aspects of signaling (e.g., oxidative signaling).

## CROSSTALK BETWEEN KAC AND OTHER PTM

As yet we have only a nascent understanding and appreciation of the complexity of various interacting PTM (van Noort et al., 2012; Rao et al., 2013). Decoding the various levels of crosstalk patterns is critical to appreciating the role of PTM in protein regulation, signaling and plant development, and controlling gene expression. The possibilities are manifold, and include multiple instances of the same PTM at different sites [e.g., a priming modification at site A is necessary for subsequent phosphorylation or acetylation of site B (Lu et al., 2011; Woodsmith et al., 2013)], to hierarchical responses to multiple PTM of the same site (Minguez et al., 2012; Zauner et al., 2012), differential responses to multiple different PTM at different sites within the same protein (cis-crosstalk), and ultimately to crosstalk between PTM of different proteins (trans-crosstalk).

An instance of PTM cis-crosstalk is the phosphorylation of Ser10 residue of histone H3, which subsequently leads to acetylation of Lys14 residue (Roth et al., 2001). The amino- and carboxy-terminal tails of the core histones are decorated with multisite-modifications including methylation, acetylation, phosphorylation, ADP-ribosylation, ubiquitination, and sumoylation (Lau and Cheung, 2013). The “histone code” was introduced as an explanation of how combinatorial systems of histone PTM regulate transcription (Jenuwein and Allis, 2001). The histone-code hypothesis was subsequently modified, extended to include transcription factors, and referred to as the PTM code (Benayoun and Veitia, 2009). With the widespread occurrence of a plethora of PTM, we propose further extension of the “PTM code” to include regulation, signaling, and development, as well as control of gene expression.

## CONCLUSION

While reversible O-phosphorylation has received the most attention of any PTM, there are an increasing number of reports of Lys-N^𝜀^-acetylation. Based upon results from analyses of mammalian systems, it is reasonable to expect discovery of many additional sites of PKA of plant and microbial proteins. It will be important to shift research emphasis from descriptive to quantitative and to determine the stoichiometry and dynamics of PKA rather than only sites of acetylation. In O-phosphorylation there are large differences between the number of kinases/client proteins, and phosphatases. In contrast, in PKA there are large differences in the numbers of acetyltransferases/deacetylases, and client proteins. The bases for these differences are obscure. Network analyses of the writers, erasers, and readers of O-phosphorylation are relatively well-developed in contrast to corresponding network analyses of N-acetylation. In the absence of any apparent candidates for KAT in mitochondria, plastids or peroxisomes, how and where are proteins resident within these organelles Lys-acetylated? A very complex and multifaceted question addresses the nature and extent of PKA crosstalk with other PTM. Finally, it will be important to achieve an improved understanding of the roles of PKA in the long-range signaling pathways.

## Conflict of Interest Statement

The authors declare that the research was conducted in the absence of any commercial or financial relationships that could be construed as a potential conflict of interest.

## Footnotes

1 http://www.uniprot.org/docs/ptmlist

2 http://p3db.org

3 http://scop.mrc-lmb.cam.ac.uk/scop/

## ABBREVIATIONS

BRD: bromodomains
HAT: histone acetyltransferase
ISD: intrinsic sequence disorder
KAC: N^𝜀^-acetyl-Lys
KAT: protein Lys-N^𝜀^-acetyltransferases
KDAC: protein Lys-N^𝜀^-deacetylases as KDACs
MS: mass spectrometry
PKA: protein Lys-N^𝜀^-acetylation
PTM: post-translational modifications.
